# Unicorn Tapestries, Horned Animals, and Prion Disease

**DOI:** 10.3201/eid1006.AC1006

**Published:** 2004-06

**Authors:** Polyxeni Potter

**Affiliations:** * Centers for Disease Control and Prevention, Atlanta, Georgia, USA

**Keywords:** prion diseases, horned animals, unicorn tapestries, cover text, about the cover

**Figure:**
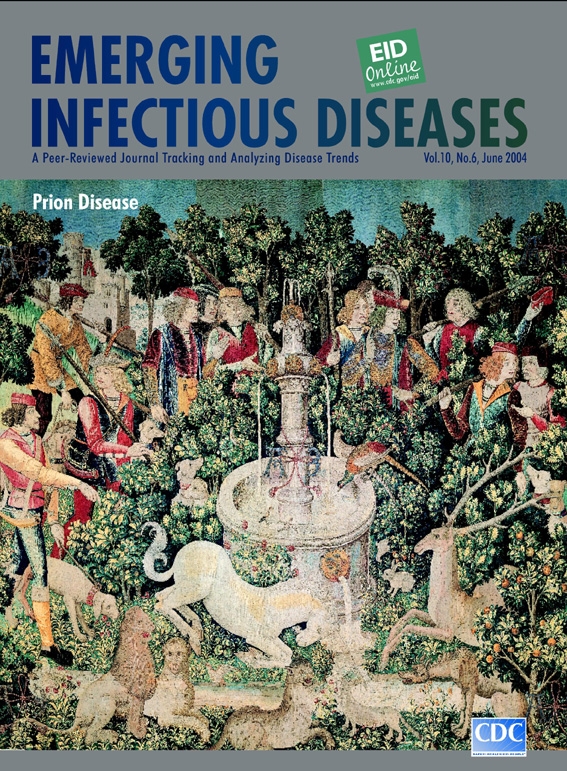
**South Netherlandish, The Unicorn Is Found, from Hunt of the Unicorn (1495–1505).** Wool warp, wool, silk, silver and gilt wefts, 368 cm x 379 cm The Metropolitan Museum of Art, Gift of John D. Rockefeller Jr., 1937 (37.80.2) Cover topic: Prion Disease

"The written text is a recent form of textile, ancillary to those primary texts 'told' or 'tooled' in cloth" (*1*). Thousands of years before humans could write, they could weave, turning cloth into a commodity, symbol of wealth, and decorative art. In many cultures, weaving has become a common linguistic metaphor as "weave" is used broadly to mean "create." A weaver "not only fashions textiles but can, with the same verb, contrive texts" (*2*).

Textiles, which date back to the Neolithic and Bronze Ages, have strong egalitarian roots and an ancient connection with personal expression (*3*). The primary purpose of weaving was storytelling. In Greek mythology, skilled Arachne challenged Athena, the goddess of wisdom and patron of the loom, to a contest. Arachne wove an elaborate account of the scandalous lives of gods and won respect from the judges but wrath from Athena, who turned her into a spider. In 11th-century France, unknown weavers stitched the conquest of England on 70 meters of linen in the Bayeux Tapestry.

Countless techniques and styles of weaving evolved throughout the ages, producing imaginative designs through a tactile process (*4*). In the Middle Ages, representational and other scenes commissioned by wealthy patrons and composed in tapestry workshops all over Europe decorated and insulated large structures (*5*). Luxurious wool, silk, and other threads were used in massive tapestries depicting love of nature and the outdoors; a penchant for luxury; and a desire to communicate, shown in banners, initials, and other messages encrypted on the scenes.

The Unicorn Is Found, on this month's cover of Emerging Infectious Diseases, is one of seven famed tapestries depicting the Hunt of the Unicorn. The works are housed at The Cloisters, a part of the Metropolitan Museum of Art, New York, designed specifically to evoke the context in which these and other medieval artifacts were created (*6*). The brilliant descriptions in these tapestries of exotic plants and animals, local customs, and human endeavor document a thriving arts scene in an era often labeled the "Dark Ages."

The tapestries, whose origins and history are as mysterious and enigmatic as their designs, could have belonged to Anne of Brittany, who married King Louis XII in 1499 (*6*). All tapestries prominently display the cipher AE and have a unifying theme: the struggle between humans and the unicorn. This creature, whose mythologic origins go back to ancient China, dominates the scenes and not only for its unique phenotypic demeanor. Strong and purposeful, it claims center stage, interceding on behalf of animals and working for the common good—purifying the stream. Although the motives and intentions of the medieval weavers and their patrons are unknown, the tapestries are likely allegorical. Why else would a beautiful, light-hearted, and benign creature on a positive mission be hunted like a common stag?

The Unicorn Is Found is thick with foliage, animals, and hunters crowding around a fountain to observe the outlandish creature. Woven in minute detail, the gothic landscape captures a universal fascination with myth and magic. As the horn is dipped into the stream, the "hunters," decked out in finery inappropriate for the hunt, form a conspiratorial circle, plotting the kill. Hunting-dogs afoot, reason aside, they close in. No one knows where the madness started or how it spread. Tension mounts as animals, beneficiaries of the unicorn's gesture, gather sheepishly in the foreground, anticipating its capture and fall. The color is intense, the crowd fickle, the unexplained madness infectious.

As travel to remote parts of the globe put myths and legends first under scrutiny and then to rest, unicorn sightings have ceased. Less exotic horned creatures, among them deer, elk, mule deer, greater kudu, have persevered in spite of at times heavy hunt and an array of infections, including, recently, prion diseases. Chronic wasting disease, which spreads by unknown routes among susceptible deer and elk, is on the increase (*7*). Like the madness in Hunt of the Unicorn and bovine spongiform encephalopathy (BSE) in other mammals, this disease could break the species barrier through foodborne or other transmission and extend its devastating neurologic effects to humans. Of all species naturally exposed to BSE, the greater kudu, perhaps the exotic unicorn's closest relative on the tapestry scene, appears most susceptible (*8*).

## References

[1] Kruger KS. Weaving the word: the metaphorics of weaving and female textual production. Selinsgrove (PA): Susquehanna University Press; 2001.

[2] Kathryn Sullivan Kruger [cited 2004 April]. Available from: http://www.susqu.edu/su_press/Q&A/KrugerQ&A.htm

[3] Barber E. Prehistoric textiles: the development of cloth in the Neolithic and Bronze Ages with special reference to the Aegean. Princeton (NJ): Princeton; 1991.

[4] Mather AD. The art of rug hooking. New York: Sterling Publishing Co.; 1998.

[5] European Tapestry Production [cited 2004 April]. Available from: http://www.metmuseum.org/toah/hd/taps/hd_taps.htm

[6] Hibbard H. The Metropolitan Museum of Art. London: John Calmann and Cooper Ltd; 1980.

[7] Belay ED, Maddox RA, Williams ES, Miller MW, Gambetti P, Schonberger NB. Chronic wasting disease and potential transmission to humans. Emerg Infect Dis. 2004;10. 15207045 10.3201/eid1006.031082 PMC3323184

[8] Cunningham AA, Kirkwood JK, Dawson M, Spencer YI, Green RB, Wells GAH. Distribution of bovine spongiform encephalopathy in greater kudu (*Tragelaphus strepsiceros*). Emerg Infect Dis. 2004;10. 15207051 10.3201/eid1006.030615 PMC3323176

